# DPNN-ac4C: a dual-path neural network with self-attention mechanism for identification of N4-acetylcytidine (ac4C) in mRNA

**DOI:** 10.1093/bioinformatics/btae625

**Published:** 2024-10-17

**Authors:** Jiahao Yuan, Ziyi Wang, Zhuoyu Pan, Aohan Li, Zilong Zhang, Feifei Cui

**Affiliations:** School of Computer Science and Technology, Hainan University, Haikou 570228, China; School of Computer Science and Technology, Hainan University, Haikou 570228, China; International Business School, Hainan University, Haikou 570228, China; Graduate School of Informatics and Engineering, The University of Electro-Communications, Tokyo 182-8585, Japan; School of Computer Science and Technology, Hainan University, Haikou 570228, China; School of Computer Science and Technology, Hainan University, Haikou 570228, China

## Abstract

**Motivation:**

The modification of N4-acetylcytidine (ac4C) in RNA is a conserved epigenetic mark that plays a crucial role in post-transcriptional regulation, mRNA stability, and translation efficiency. Traditional methods for detecting ac4C modifications are laborious and costly, necessitating the development of efficient computational approaches for accurate identification of ac4C sites in mRNA.

**Results:**

We present DPNN-ac4C, a dual-path neural network with a self-attention mechanism for the identification of ac4C sites in mRNA. Our model integrates embedding modules, bidirectional GRU networks, convolutional neural networks, and self-attention to capture both local and global features of RNA sequences. Extensive evaluations demonstrate that DPNN-ac4C outperforms existing models, achieving an AUROC of 91.03%, accuracy of 82.78%, MCC of 65.78%, and specificity of 84.78% on an independent test set. Moreover, DPNN-ac4C exhibits robustness under the Fast Gradient Method attack, maintaining a high level of accuracy in practical applications.

**Availability and implementation:**

The model code and dataset are publicly available on GitHub (https://github.com/shock1ng/DPNN-ac4C).

## 1 Introduction

The ac4C RNA modification is highly conserved across prokaryotes and eukaryotes. Facilitated by the ac4C modification enzyme, N4-acetylcytidine undergoes acetylation at the N4 position. This modification extends beyond eukaryotic tRNA and 18S rRNA, also manifesting in mammalian mRNA. It serves to enhance translation efficiency, increase mRNA stability, and regulate gene expression (Arango *et al.* 2018, Zhang *et al.* 2023). NAT10 (N-acetyltransferase 10) currently stands as the sole known eukaryotic enzyme responsible for ac4C modification, with its catalyzed mRNA implicated in various human diseases, notably cancer (Luo *et al.* 2023).

Research by Yan *et al.* validated that NAT10-mediated RNA ac4C modification induces lytic replication of the DNA virus Kaposi’s sarcoma-associated herpesvirus (KSHV) (Yan *et al.* 2023). Furthermore, Zong *et al.* reported an ac4C-dependent modification of mRNA that elevates AXL expression, contributing to enhanced metastasis in Pancreatic Ductal Adenocarcinoma (PDAC) (Zong *et al.* 2023). Notably, NAT10 has been identified as a promoter of cancer metastasis and epithelial-mesenchymal transition (EMT) through ac4C modification of COL5A1 mRNA (Zhang *et al.* 2021). These findings underscore the significance of recognizing ac4C modifications on mRNA for disease detection, gaining a profound understanding of their translation mechanisms, and early prevention.

In recent years, studies have demonstrated that the detection of RNA ac4C can be achieved through biotechnological approaches. For instance, quantitative single-nucleotide resolution mapping of cytidine acetylation in RNA, known as ac4C sequencing (ac4C-seq) (Thalalla Gamage *et al.* 2021), liquid chromatography-mass spectrometry (LC-MS) based on ac4C-seq (Thalalla Gamage *et al.* 2021), and the high-throughput sequencing method acRIP-seq proposed by Daniel Arango *et al.* (Zhao *et al.* 2019).

While these biotechnological approaches effectively detect ac4C modifications in RNA, their implementation requires specialized instruments, domain experts, and is both time-consuming and costly. In the past few years, the development of machine learning has made it possible to devise more efficient and cost-effective methods for RNA ac4C sequencing. PACES, proposed by Zhao *et al.* integrates two random forest classifiers, namely, position-specific dinucleotide sequence profiles and K-nucleotide frequencies. This method utilizes genomic sequences as input, and based on the training model, PACES provides potential modified sequences (Zhao *et al.* 2019). Subsequently, Alam *et al.* introduced the XG-ac4C machine learning model based on the extreme gradient boosting classifier for identifying ac4C sites. The XG-ac4C model further enhances its classification performance by incorporating the combination of the electron-ion interaction pseudo-potential and the electron-ion interaction pseudo-potential of the three-nucleotide sequence in ac4C sites (Alam *et al.* 2020). Wang *et al.* proposed DeepAc4C, a convolutional neural network (CNN)-based model for identifying ac4C in mRNA. DeepAc4C integrates physicochemical patterns and distributed representation information of nucleic acids, enhancing predictive performance through feature optimization and dimensionality reduction (Wang *et al.* 2021). Su *et al.* proposed a novel prediction model named iRNA-ac4C based on three encoding methods: nucleotide composition, nucleotide chemical property, and accumulated nucleotide frequency. Integrating mRMR feature selection and GBDT, iRNA-ac4C demonstrates superior performance in identifying ac4C sites compared to previous models (Su *et al.* 2023). Pham *et al.* proposed the ac4C-AFL method, which uses an innovative ensemble feature importance scoring (EFIS) strategy, enabling ac4C-AFL to extract the most representative features from RNA sequences. Utilizing these features, researchers constructed 176 baseline models and applied a two-step feature selection method, combined with a support vector machine (SVM) classifier, to develop the final predictive model (Pham *et al.* 2024).

While previous models have made significant strides, they often leaned heavily on manual feature extraction and traditional machine learning methods (Cui *et al.* 2021b, 2022b). Deep learning techniques, in contrast to traditional models, provide a notable advantage in the more profound and efficient extraction of local information within features (LeCun *et al.* 2015, Jiao *et al.* 2023). This advantage is particularly well-suited for tasks such as identifying ac4C sites in RNA. Moreover, the integration of manual feature extraction with deep learning approaches has seldom been explored in the realm of ac4C recognition tasks.

**Figure 1.**
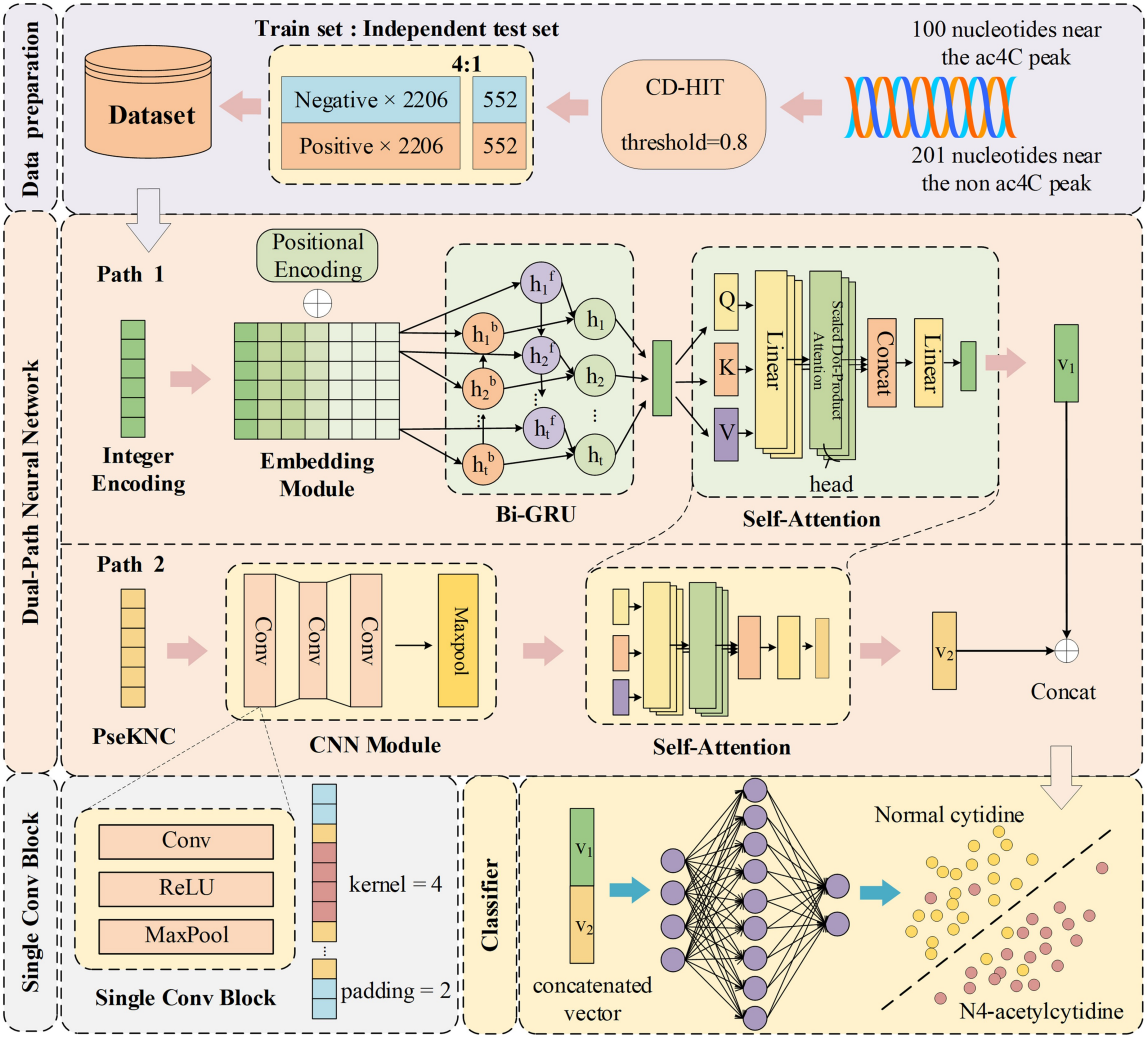
Main framework of the model. The first layer represents the data preparation section. The second layer provides a detailed description of the model. On the third layer, the details of single conv block are depicted on the left, while the classifier diagram is shown on the right.

We address the aforementioned concerns by proposing a dual-path neural network utilizing a self-attention mechanism, featuring two distinct neural network structures. Main framework of the model is shown in Fig. 1. The first path incorporates an Embedding layer, a bidirectional GRU network, and a self-attention mechanism. Meanwhile, the secondary path is predominantly comprised of a CNN network and a self-attention mechanism. Within this framework, the Embedding layer facilitates the acquisition of a higher-dimensional representation of RNA features, the bidirectional GRU network adeptly captures contextual information from RNA sequences (Chung *et al.* 2014), fostering a comprehensive understanding of interactions among ribonucleotides. Simultaneously, the CNN network comprehensively extracts local features from sequence characteristics, particularly aiding in the identification of ac4C modification (Zeng *et al.* 2016, Cui *et al.* 2022a). Furthermore, the self-attention mechanism excels in capturing long-range dependencies within RNA sequences (Vaswani *et al.* 2017).

Based on 10-fold cross-validation, our model in this study outperforms the state-of-the-art models. It achieves higher accuracy of 83.89%, MCC of 66.55%, AUROC of 92.72%, and SPE of 85.6%. To assess the model’s robustness, we introduced the Fast Gradient Method (FGM) (Jin *et al.* 2022) module to attack the network. Even after the attack, our model maintains an accuracy of 83.72%.

## 2 Materials and methods

### 2.1 Benchmark dataset

To facilitate comparison with prior models, we adopted the dataset consistent with the approach proposed by Su *et al.* (2023). To establish a reliable dataset, researchers identified cytidines closest to ac4C peaks as modification sites. Subsequently, 100 nucleotides were chosen on both sides of these modification sites to create positive samples. Negative samples were randomly selected from nonpeak regions, with their sequences matching the length of positive samples (201 nucleotides), and the central sites being cytidine. To address concerns about redundant sequences impacting prediction performance, the CD-HIT (Li and Godzik 2006) tool was utilized to eliminate similar sequences, using a threshold of 0.8. Creating a balanced dataset involved randomly selecting an equal number of sequences from negative samples as from positive samples. Following these procedures, the positive and negative data were randomly divided into a training dataset and an independent testing dataset at a 4:1 ratio. The final training dataset comprised 2206 positive samples and 2206 negative samples, while the independent dataset consisted of 552 positive samples and 552 negative samples.

### 2.2 Feature encoding schemes

After preparing the data, the subsequent step involves designing and extracting feature vectors. In recent years, diverse feature encoding strategies have been used to represent biological sequences like protein, DNA and RNA, yielding remarkable outcomes (Cui *et al.* 2021a, Ao *et al.* 2022, Wang *et al.* 2023). Notable examples include PseKNC (Guo *et al.* 2014, Chen *et al.* 2015, Su *et al.* 2021), one-hot encoding (Chen *et al.* 2020, Yuan *et al.* 2022, Zulfiqar *et al.* 2022), integer encoding (Cao *et al.* 2023), and word2vec features (Dao *et al.* 2021, Lv *et al.* 2021). For this study, we adopt the widely utilized PseKNC and integer encoding techniques to represent RNA fragments. The following section offers a comprehensive explanation of these two encoding approaches.

#### 2.2.1 Pseudo K-tuple nucleotide composition (PseKNC)

PseKNC, short for Pseudo K-tuple Nucleotide Composition, serves as a technique for extracting features from DNA, RNA, or protein sequences. Its primary purpose is to delineate the distribution of k-mers within biological sequences, facilitating a more precise capture of local structural information. Initially, a given RNA sequence S, comprising L bases, undergoes segmentation into nonoverlapping subsequences of length k, termed as k-mers. K-tuple Nucleotide Composition (KNC) is used to depict the combinations and frequencies of these k-mers. The occurrences of N types of k-mer within the sequence are tallied, resulting in the construction of a frequency distribution vector, denoted as$\left\{X_{1},X_{2},\ldots ,X_{N}\right\}$. Subsequently, the frequency of each k-mer in the sequence is computed to derive a frequency vector:

(1) $$\vec{P}=\left(P_{1},P_{2},\ldots ,P_{N}\right).$$

Here, $P_{i}$ represents the frequency of the i-th type of k-mer. Secondly, PseKNC enhances the expression capability of sequence features by introducing “pseudo-components.” This encompasses two aspects of pseudo-components: one derived from the weight factor $\omega_{i}$ of each k-mer frequency:

(2) $$\vec{W}=\left(\omega_{1},\omega_{2},\ldots ,\omega_{N}\right).$$

The second aspect involves a correlation vector derived from the correlation between different k-mers:

(3) $$\vec{R}=\left(r_{1},r_{2},\ldots ,r_{N}\right).$$

Finally, the frequency vector, weight factors, and correlation factors of KNC are combined to obtain the PseKNC feature vector:

(4) F→=P→,W→,R→.

Illustrating with a simple example using k-mer = 2: Assuming the input RNA sequence is: AGCUUAG.

Firstly, spliting the RNA sequence into 2-mers, obtaining: AG, GC, CU, UU, UA, AG. Then, we calculate the frequency of each 2-mer: {“AG”: 2, “GC”: 1, “CU”: 1, “UU”: 1, “UA”: 1}. Thus, we obtain:

(5) $$\vec{P}=\left(2,1,1,1,1\right).$$

Next, we introduce weight and correlation factors. Here, assuming each 2-mer has the same weight, it can be represented as {“AG”: 2, “GC”: 1, “CU”: 1, “UU”: 1, “UA”: 1}. Thus, we obtain:

(6) $$\vec{W}=\left(1,1,1,1,1\right).$$

Finally, introducing the correlation factor. Here, using the mean and standard deviation, it calculates to {“mean”: 1.5, “std”: 0.5}. Based on this data, we obtain:

(7) $$\vec{R}=\left(1.5,0.5\right).$$

Ultimately, calculating:

(8) F→=P→,W→,R→=2,1,1,1,1+1,1,1,1,1+1.5,0.5=3,2,2,2,2,1.5,0.5.

#### 2.2.2 Integer encoding of sequences

In this study, we utilize Integer Encoding as the second method for encoding mRNA sequences. Integer encoding maps RNA sequence bases to integers. Integrated with a bidirectional GRU network, Integer Encoding effectively analyzes contextual relationships within sequences. Notably, it intuitively represents positional order relationships between bases, complementing the GRU network’s sequence analysis capabilities. The implementation process involves defining a base dictionary where each symbol corresponds to a discrete integer value. Let RNA sequence S with L bases be denoted as $S=R_{1}R_{2}R_{3}{\ldots R}_{L}$, where $R_{i}=\{A,U,C,G\}$. By systematically mapping A to 0, C to 1, G to 2, and U to 3, we obtain the final sequence encoding. Using the RNA sequence AGCUUAG as an example, after applying the mapping relationship provided in the table, we obtain [0, 3, 2, 1, 1, 0, 3].

### 2.3 Model architecture of DPNN-ac4C

The DPNN-ac4C model comprises two main paths. The first path comprises an Embedding module, bidirectional GRU network, and self-attention mechanism. The Embedding module is primarily designed by referencing the embedding combination approach of transformers (Vaswani *et al.* 2017). Since the input to the Embedding layer is an integer-encoded numerical array, similar to the WordPiece (Devlin *et al.* 2018) method of BERT’s tokenizer, features are mapped to higher-dimensional representations in the Embedding layer. The bidirectional GRU network further learns deeper semantic information, while the self-attention mechanism emphasizes features related to ac4C sites. The second path integrates a CNN module and self-attention mechanism. The CNN network extracts deeper insights about ac4C sites from PseKNC features, while the self-attention module enhances focus on relevant parts of the sequence. The source code of the model can be obtained for free on GitHub: https://github.com/shock1ng/DPNN-ac4C.

#### 2.3.1 Embedding module

In this module, features undergo integer encoding before entering the Embedding layer. This layer converts the features into higher-dimensional vectors, which are adaptable and evolve during model training to enhance the original feature representation (Li *et al.* 2022). After this transformation, positional encoding (Vaswani *et al.* 2017) further strengthens the positional information representation. The detailed steps of positional encoding are outlined below:

Let $v\in R^{L\times d_{k}}$ denote the vector output after passing through the Embedding layer, and $PE\in R^{L\times d_{k}}$ represent the positional encoding matrix. The positional encoding matrix PE computes its odd and even columns separately, as shown in Equations (9)–(11):

Odd columns:

(9) $$PE=\cos\left(e^{-\frac{x\mathrm{lo}g^{a}}{d_{k}}}\right).$$

Even columns:

(10) $$PE=\sin\left(e^{-\frac{x\mathrm{lo}g^{a}}{d_{k}}}\right).$$

Vector encoded by positional encoding (such as formula 7):

(11) $$v_{\mathrm{embedding}}=v+PE.$$

Where $x\in [0,\frac{d_{k}}{2}]$, a∈N.

#### 2.3.2 Bidirectional GRU network (Bi-GRU)

The bidirectional GRU network, a variant of recurrent neural networks (RNNs), is commonly used for sequential tasks. “Bidirectional” implies that the network considers both past and future information at each time step. GRU, or Gated Recurrent Unit, effectively captures long-term dependencies within sequences and offers higher training efficiency compared to LSTM due to fewer parameters. By using two independent GRUs—one processing input from left to right (forward) and the other from right to left (backward)—Bi-GRU comprehensively models input sequences bidirectionally, making it widely applicable in fields like natural language processing and bioinformatics (Min *et al.* 2017, Nosouhian *et al.* 2021). Our Bi-GRU model, illustrated in Fig. 1, primarily consists of forward hidden states $h_{t}^{f}$, backward hidden states $h_{t}^{b}$, and outputs $h_{t}$ at time step t, computed using Equations (12)–(14):

(12) $$h_{t}^{f}=\left(1-z_{t}^{f}\right)⊙h_{t-1}^{f}+z_{t}^{f}⊙\tanh\left(W_{hx}^{f}\cdot x_{t}+b_{hx}^{f}+r_{t}^{f}⊙W_{hh}^{f}\cdot h_{t-1}^{f}+b_{hh}^{f}\right),$$

(13) $$h_{t}^{b}=\left(1-z_{t}^{b}\right)⊙h_{t+1}^{b}+z_{t}^{b}⊙\tanh\left(W_{hx}^{b}\cdot x_{t}+b_{hx}^{b}+r_{t}^{b}⊙W_{hh}^{b}\cdot h_{t+1}^{b}+b_{hh}^{b}\right),$$

(14) $$h_{t}=\left[h_{t}^{f},h_{t}^{b}\right],$$

where $x_{t}$ represents the features at time step t of the input sequence, $z_{t}^{f}$ and $z_{t}^{b}$ are update gates, $r_{t}^{f}$ and $r_{t}^{b}$ are reset gates, W denotes weights, b denotes biases, and ⊙ denotes element-wise multiplication. The superscript f denotes the forward GRU, while b denotes the backward GRU. The formulas for the update and reset gates are as follows (Equations 15–18):

(15) $$z_{t}^{f}=\sigma \left(W_{zh}^{f}\cdot h_{t-1}^{f}+W_{zx}^{f}\cdot x_{t}+b_{zh}^{f}+b_{zx}^{f}\right).$$

(16) $$z_{t}^{b}=\sigma \left(W_{zh}^{b}\cdot h_{t+1}^{b}+W_{zx}^{b}\cdot x_{t}+b_{zh}^{b}+b_{zx}^{b}\right).$$

(17) $$r_{t}^{f}=\sigma \left(W_{rh}^{f}\cdot h_{t-1}^{f}+W_{rx}^{f}\cdot x_{t}+b_{rh}^{f}+b_{rx}^{f}\right).$$

(18) $$r_{t}^{b}=\sigma \left(W_{rh}^{b}\cdot h_{t+1}^{b}+W_{rx}^{b}\cdot x_{t}+b_{rh}^{b}+b_{rx}^{b}\right).$$

Here, σ represents the sigmoid activation function, whose formula is given by (Equation 19):

(19) $$\sigma \left(x\right)=\frac{1}{1+e^{-x}}.$$

The output of the sigmoid function ranges between 0 and 1, making it suitable for generating probability-based outputs and regulating the flow of information in gating units. The forward hidden state $h_{t}^{f}$, backward hidden state $h_{t}^{b}$, and output $h_{t}$ at time t collaborate to enable the bidirectional GRU network to handle sequential tasks more effectively.

#### 2.3.3 Convolutional neural network module (CNN module)

The second path in this model begins with the CNN module, a deep learning architecture widely used for data processing, notably in image and sequence analysis (Lan *et al.* 2018). CNN’s key strength lies in its ability to efficiently extract local features through convolutional layers, thereby facilitating effective learning and representation of data. To augment the network’s expressiveness, robustness, and reduce parameter count, researchers often integrate activation and pooling layers between multiple convolutional layers (Zhang and Kabuka 2020). These combined layers constitute the CNN module in this study.

Illustrated in the lower section of Fig. 1, our CNN module comprises three sub-convolutional blocks, each housing a convolutional layer, activation layer, and pooling layer (as illustrated in the bottom left corner of Fig. 1).

Let the input feature be x, and the output after a single convolutional block be x’. The formula for a single convolutional block is as follows [Equation (20)]:

(20) $$x’=\mathrm{maxpool}\left(\sigma \left(\mathrm{Conv}\left(x\right)\right)\right).$$

Here, σ represents the ReLU activation function.

After traversing three sub-convolutional blocks and a subsequent max-pooling layer, the parameters are substantially reduced, efficiently extracting the main features of the input data. This, in turn, lessens the computational load of subsequent self-attention mechanisms and mitigates the risk of overfitting.

#### 2.3.4 Multi-head self-attention module

In both paths of this model, a multi-head self-attention mechanism is utilized, integrated with both the CNN and Bi-GRU network modules. Self-attention is a fundamental mechanism in deep learning, commonly applied in Deep Learning (Vaswani *et al.* 2017, Devlin *et al.* 2018, Zhou *et al.* 2024). Unlike single attention mechanisms, which evaluate input sequences from a singular viewpoint, multi-head self-attention uses multiple parallel attention “heads,” each focusing on distinct subspaces of the input sequence through separate linear transformations (Fu *et al.* 2024). By independently computing these attention weights and then combining the results of each head, the model can concurrently capture various patterns and contextual information within the sequence data. Unlike independent Bi-GRU or CNN, which rely on fixed-length context windows or local receptive fields, self-attention mechanisms can globally process sequence data, facilitating direct connections between any two elements in the sequence. Specifically, CNN is adept at extracting local patterns of consecutive nucleotides, while Bi-GRU captures bidirectional temporal dependencies. The self-attention module further augments the capabilities of these two models, yielding the final representations $v_{1}$ and $v_{2}$ (as illustrated in Fig. 1), which not only retain local features but also augment global contextual information. This is pivotal for comprehending intricate RNA secondary structures and predicting their potential biological functions. Various researchers may use different algorithms for self-attention. In the model (as illustrated in Fig. 1), the formula for multi-head self-attention is as follows (Equations 21–24):

Assuming the input vector into the self-attention mechanism is:

(21) Q=xWQK=xWKV=xWV.

(22) $$\mathrm{Attention}(Q,K,V)=\mathrm{Softmax}\left(\frac{QK^{T}}{\sqrt{d_{k}}}\right)V.$$

(23) $${\mathrm{head}}_{i}=\mathrm{Attention}\left(Q_{i},K_{i},V_{i}\right).$$

(24) $$\mathrm{MultiHead}\left(Q,K,V\right)=\mathrm{Concat}\left({\mathrm{head}}_{1},{\mathrm{head}}_{2}{,\ldots ,\mathrm{head}}_{h}\right)W^{o}.$$

The dimensions of the mapping matrices here are respectively: $W^{Q}\in R^{d_{\mathrm{model}}\times d_{k}}$, $W^{K}\in R^{d_{\mathrm{model}}\times d_{k}}$, $W^{V}\in R^{d_{\mathrm{model}}\times d_{k}}$, and $W^{o}\in R^{{{hd}_{v}\times d}_{\mathrm{model}}}$. In this model, we use a number of heads h=8, and $d_{k}=d_{v}=d_{\mathrm{model}}/h=64$.

#### 2.3.5 Deep neural network classifier (DNN classifier)

In this study, we utilize a DNN classifier for the final classification task. The input to this classifier is the concatenation of outputs from the preceding two network paths, aiming to integrate features from different models. Deep neural networks have robust feature learning capabilities, allowing them to extract deep, abstract RNA class features from concatenated high-dimensional features, aiding in discerning subtle sequence differences and hidden biochemical characteristics. Denoting the vector input to the DNN as x, the formula for the DNN model is represented by Equation (25):

(25) $$y=\mathrm{Softmax}(W_{2}\cdot \sigma (W_{1}x+b_{1})+b_{2}).$$

Here, σ represents the ReLU activation function, W denotes the matrix transformation parameters of the input, and b represents the bias term.

### 2.4 Fast gradient method (FGM) robustness detection method

Due to the possibility of errors during RNA sequencing, and these errors are often difficult to avoid. To assess the model’s robustness, we utilized the FGM module to attack the network. Using FGM in this RNA classification task involves creating precise perturbations to the model by adjusting the feature values of input RNA sequences, aiming to induce erroneous predictions. The detailed implementation is outlined in Equation (26):

(26) δ=ε⋅sign(∇xJ(θ,x,y))xattack=x+δ.

Where x denotes the input vector, y represents the corresponding label vector, and J symbolizes the loss function, which cross-entropy is used. ε is a constant, denoting the perturbation magnitude or attack budget. The sign(·) function yields a positive or negative sign. Adversarial samples generated are noted $x_{\mathrm{attack}}$.

### 2.5 Performance evaluation strategies

This study evaluates the model using five commonly used metrics in machine learning, namely accuracy (ACC), area under the receiver operating characteristic curve (AUROC), Matthews correlation coefficient (MCC), specificity (SPE), and sensitivity (SEN). The formulas for these metrics are as follows [Equation (27)]:

(27) ACC=TP+TNTP+TN+FP+FNAUROC=∫TPRdFPRMCC=TP×TN-FP×FNTP+FPTP+FNTN+FPTN+FNSPE=TNTN+FPSEN=TPTP+FN.

In the above formulas, True Positive (TP) represents the number of correctly predicted positive samples, while True Negative (TN) represents the number of correctly predicted negative samples. False Positive (FP) indicates the number of incorrectly predicted positive samples, and False Negative (FN) indicates the number of incorrectly predicted negative samples. ACC, or accuracy, is one of the most intuitive and commonly used evaluation metrics, representing the proportion of correctly classified samples to the total number of samples. A high accuracy implies overall good predictive performance across all classes. MCC is a statistic used for both binary and multiclass classification problems, particularly suitable for imbalanced classes. It considers four cases: True Positive (TP), False Positive (FP), True Negative (TN), and False Negative (FN). MCC is a comprehensive measure with values ranging from −1 to 1, where 1 indicates perfect agreement, 0 implies predictions unrelated to the actual results, and −1 represents completely opposite predictions. AUROC curve depicts the global assessment of the model’s ability to distinguish between positive and negative samples. The AUC value is the area under the ROC curve, ranging from 0 to 1. A higher AUC value indicates a stronger ability of the model to discriminate between positive and negative samples, while 0.5 represents random guessing. SPE, or true negative rate, refers to the proportion of samples correctly identified as negative among all actual negative samples. In tasks like disease diagnosis, specificity reflects the model’s ability to exclude nondisease cases. SEN, or recall rate, refers to the proportion of samples correctly identified as positive among all actual positive samples. In the field of bioinformatics, sensitivity reflects the accuracy of the model in identifying RNA modification sites, disease-positive samples, etc.

## 3 Experiments and results

To gain a more precise understanding of the impact of specific hyperparameters on the network’s recognition performance, this study explored the effects of various hyperparameter combinations on the model, without altering the original model framework. Specifically, we investigated the influence of the hidden layer size of the GRU network on the representational capacity for RNA sequences. We set the hidden layer sizes to 256, 512, and 768 dimensions and varied whether the network was bidirectional. The results, as shown in the Table 1 (bold black font indicates the maximum value), indicate that a GRU network without bidirectional capabilities has a poorer recognition ability for RNA sequences. The best performance was achieved with a hidden layer size of 512. This is because when the hidden layer is too small, the network’s extraction of information from the RNA sequence is overly condensed; conversely, when the hidden layer is too large, the extraction becomes too broad, preventing the network from accurately recognizing RNA sequences.

**Table 1.** Comparison of different hyperparameter settings in GRU based on the 10-fold cross-validation of the training set. (Bold indicates maximum value in the current column)

Hidden_state	Bi-direction	ACC (%)
256	Yes	82.41
**512**	**Yes**	**83.89**
768	Yes	83.56
256	No	82.33
512	No	82.75
768	No	82.52

To understand the representational capacity of the CNN network for RNA sequences in relation to hyperparameters, we set the convolutional kernel sizes to 3, 4, and 5, and the padding sizes to 1, 2, and 3. The results, as shown in the Table 2, suggest that adjustments to the hyperparameters of the convolutional network have a minor impact on recognition performance. However, when the convolutional kernel size is 5, the recognition accuracy is significantly lower, likely due to the large kernel size condensing the sequence representation, which degrades the network’s recognition performance.

**Table 2.** Comparison of different hyperparameter settings in CNN based on the 10-fold cross-validation of the training set. (Bold indicates maximum value in the current column)

Kernel	Padding	ACC (%)
3	1	83.53
3	2	83.78
3	3	83.44
4	1	83.74
**4**	**2**	**83.89**
4	3	83.51
5	1	82.55
5	2	83.12
5	3	82.38

After analyzing the hyperparameter choices for both GRU and CNN networks, we selected a bidirectional GRU network with a hidden layer size of 512, and set the CNN hyperparameters to a convolutional kernel size of 4 and a padding size of 2. In addition, during training, the learning rate was set to 0.001, using the Adam optimizer and cross-entropy loss. The experiments used 10-fold cross-validation, where the training dataset was divided into 10 parts. In each fold of cross-validation, one part was nonrepetitively selected as the validation set, and the remaining parts served as the training set. After each fold’s training, the parameters were saved and then reset.

### 3.1 Motif analysis

**Figure 2.**
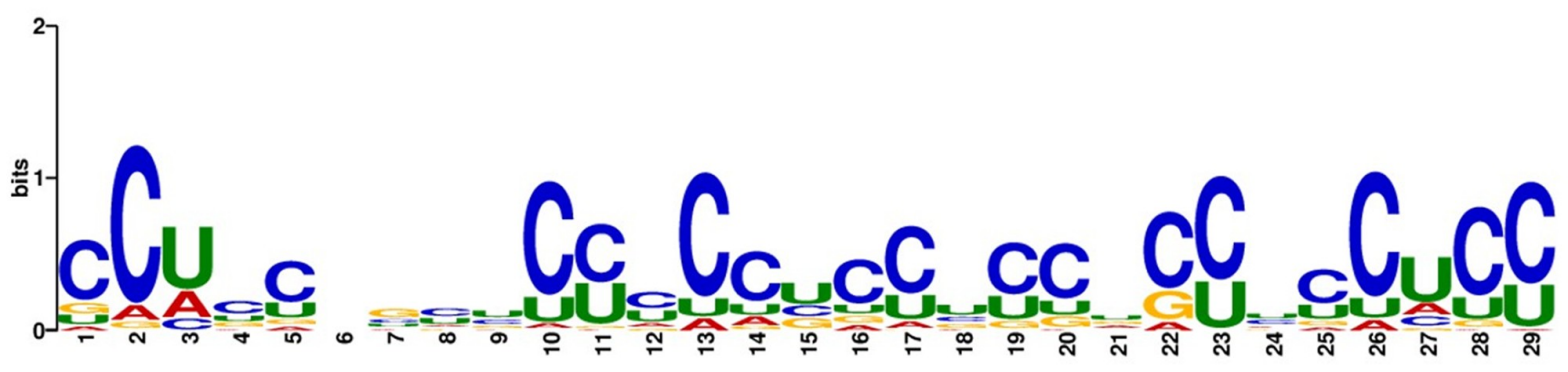
Motif analysis result of ac4C modified sequences.

To investigate the distribution and preference of nucleotides flanking ac4C peaks, we utilized the STREME (Bailey 2021) software for motif analysis, as shown in the Fig. 2. Consistent with the observations of Arango *et al.* (2018) and Su *et al.* (2023), motif identified exhibit highly significant occurrence frequencies within the sequences. Sequences containing ac4C modifications are highly enriched with the motif (CXX), a C-rich subsequence. Their research demonstrated that mRNA codons containing cytidine at the wobble site are more abundant in acetylated transcripts compared to other nonacetylated transcripts. This promotes interaction with cognate tRNAs, thereby enhancing mRNA translation efficiency. These results indicate that the sequences flanking ac4C peaks show a preference for specific modifications within coding sequences (CDS). These findings provide valuable insights for further functional studies and aid in understanding the role of these motifs in gene regulatory networks.

### 3.2 Ablation experiments for DPNN-ac4C

To understand the impact of certain components in the model on ac4C recognition predictions, we conducted ablation experiments by gradually replacing or deactivating specific parts to achieve the purpose of ablation studies. In this study, we will discuss the effectiveness of features, the importance of positional encoding, the efficacy of self-attention, and the use of FGM attacks to validate the model’s robustness. All experiments were conducted on NVIDIA’s A100 graphics card.

#### 3.2.1 Effectiveness of features

**Figure 3.**
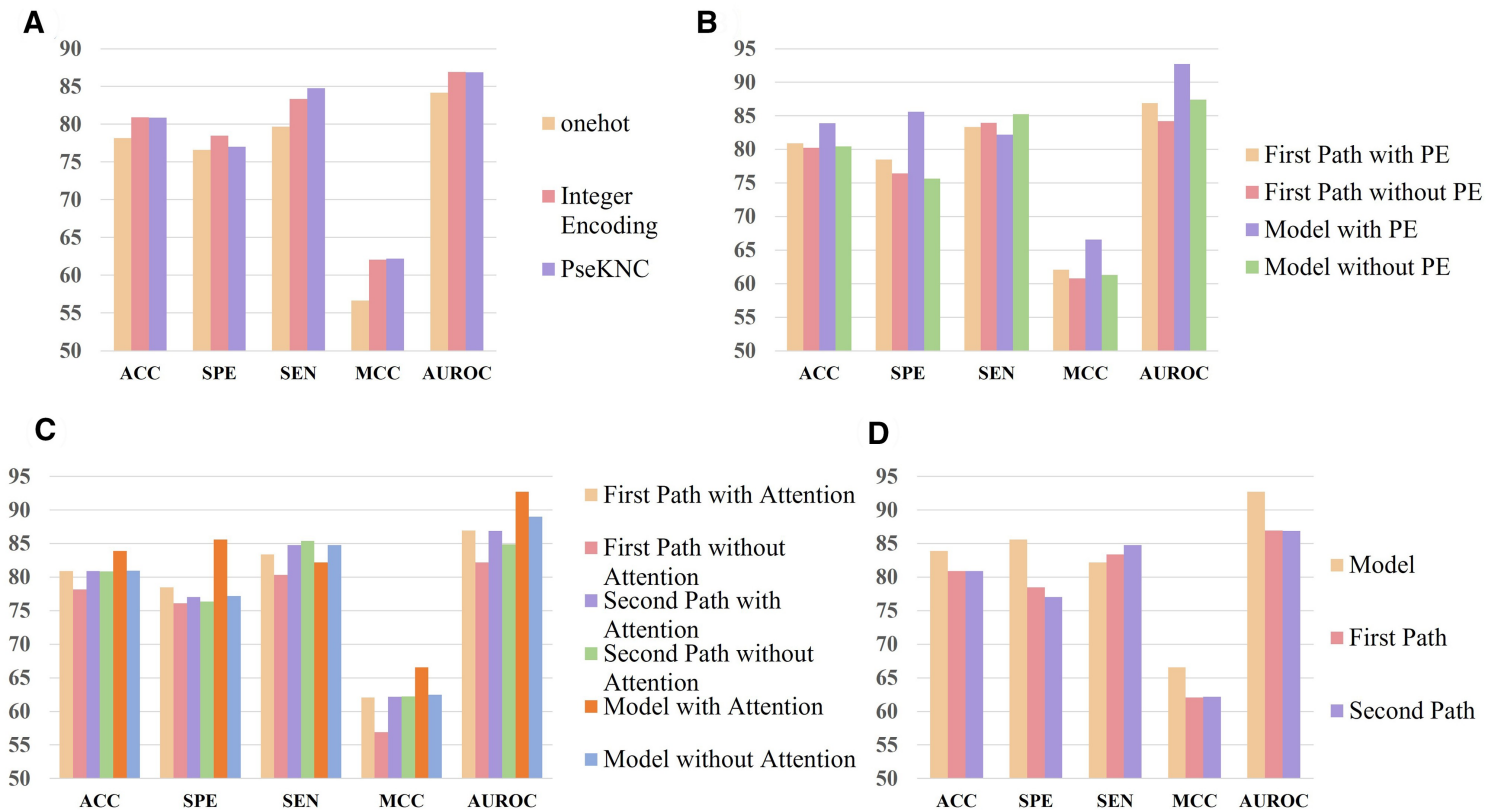
(A) Comparison of performance between one-hot encoding, sequence integer encoding, and PseKNC encoding. (B) Comparison of the impact of Positional Encoding (PE) on the model. (C) Bar chart of self-attention mechanism ablation experiment. (D) Comparison of the two paths and the model.

To validate the efficacy of Sequence Integer Encoding and PseKNC features, this study compared their performance with one-hot encoding. The performance of models using each feature individually was assessed while maintaining the model unchanged. The experimental results, depicted in Fig. 3A, demonstrate that both Sequence Integer Encoding and PseKNC features outperformed one-hot encoding. Notably, one-hot encoding achieved an accuracy of only 78.15%, whereas integer encoding and PseKNC reached 80.91% and 80.88%, respectively. In addition, the SEN, SPE, MCC, and AUROC for each encoding type were as follows: One-hot encoding: SEN 79.90%, SPE 76.61%, MCC 56.65%, AUROC 78.16%; Integer encoding: SEN 83.36%, SPE 78.48%, MCC 62.05%, AUROC 86.92%; PseKNC encoding: SEN 84.75%, SPE 77.01%, MCC 62.18%, AUROC 86.87%. Clearly, the latter two encoding methods exhibited similar model performance, surpassing one-hot encoding across all metrics. This indicates that the features utilized in this study are more effective compared to training without specific features but using deep neural networks.

#### 3.2.2 Importance of multi-head self-attention mechanism

To understand the importance of the self-attention mechanism, we conducted ablation experiments to assess its impact on model recognition. We compared the performance of the original model with a modified version lacking the self-attention mechanism. For both pathways of our model, we analyzed the differences in performance with and without the self-attention mechanism. As depicted in Fig. 3B, the first pathway showed significant improvement with the addition of the attention mechanism, with SEN, SPE, ACC, MCC, and AUROC increasing by 3.05%, 2.37%, 2.72%, 5.16%, and 4.71%, respectively. In contrast, the second pathway exhibited minimal change in performance without the self-attention mechanism, with ACC increasing by only 0.01%. However, overall model performance improved with the inclusion of self-attention, particularly evident in SPE 8.43%, ACC 2.92%, MCC 4.08%, and a remarkable 11.74% increase in AUROC. In summary, the multi-head self-attention mechanism enhanced model performance by aiding in feature weighting.

#### 3.2.3 Importance of positional encoding

To evaluate the significance of positional encoding, we conducted ablation experiments to determine its impact on model recognition. Since only the first pathway of our model used positional encoding, we separately trained this pathway without positional encoding. In addition, we presented the data of the complete model without positional encoding to illustrate its effect on recognition performance. As depicted in Fig. 3C, training the first pathway alone with positional encoding resulted in improvements in SPE 2.04%, ACC 0.68%, MCC 1.24%, and AUROC 2.71%. These findings indicate that positional encoding assists the subsequent Bi-GRU network in learning the positions of ac4C sites across time steps. Conversely, removing positional encoding from the complete model significantly reduced recognition performance, with SPE, ACC, MCC, and AUROC decreasing by 9.98%, 3.45%, 5.23%, and 5.29%, respectively. This highlights the substantial impact of positional encoding on overall model performance, underscoring its role in achieving significantly better results.

#### 3.2.4 Importance of two paths

In order to further compare the contribution of different pathways in the model, we compared the performance of the model we proposed DPNN-ac4C and its two paths. As shown in Fig. 3D, after integrating the two paths, although the overall model’s SEN index further decreased, the overall SPE is higher. Comparing the two paths, the overall model’s ACC increased by about 3%, MCC increased by about 4%, and AUROC increased by about 6%. The overall model can integrate the local features learned by PseKNC combined with CNN network and the time series features combined with integer encoding and Bi-GRU, thus showing higher performance.

#### 3.2.5 Feature combination ablation experiment

**Figure 4.**
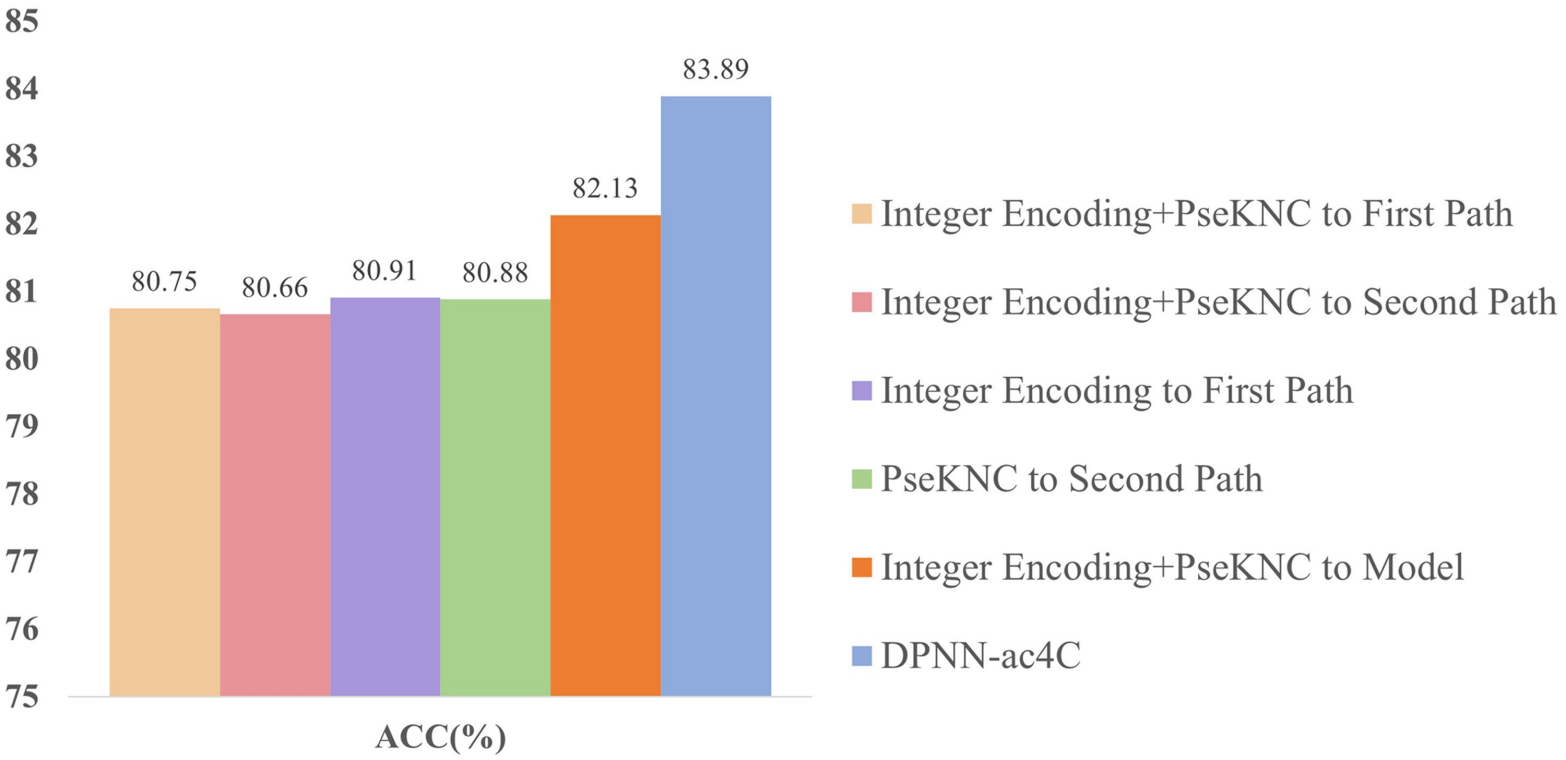
Feature combination ablation experiment bar chart, where DPNN-ac4C in the legend represents the combined effect of integer encoding entering the first path and PseKNC encoding entering the second path.

To further understand the representational capacity of features and their impact on the model’s recognition accuracy, we conducted a series of comparative experiments combining integer encoding with PseKNC. The results of the 10-fold cross-validation experiments are shown in the Fig. 4. As the figure indicates, the introduction of feature combinations brought in unnecessary dimensional vectors, and redundant features introduced a certain degree of noise to the network, increasing the computational load of the model. Although the combination of features brought some unnecessary additional feature dimensions, causing unnecessary trouble for the network, the network still maintained a high recognition accuracy, which also indirectly demonstrates the robustness of our network structure.

### 3.3 Comparison with existing models

In this study, we compared our model with three existing models: PACES (Zhao *et al.* 2019), XG-ac4C (Alam *et al.* 2020), iRNA-ac4C (Su *et al.* 2023), and DeepAc4C (Wang *et al.* 2021). These models utilize handcrafted features and rely more on traditional machine learning decision tree models for classification. To ensure a more objective experimental outcome, when comparing the experimental data, we individually reproduced and deployed the aforementioned models on the training server, performing 10-fold cross-validation on the training set and then comparing their performance on an independent test set. In addition, since the ac4C-AFL method does not provide the source code, a direct comparison with this method is not possible. The results of 10-fold cross-validation on the training set are summarized in Table 3. Among the existing models, iRNA-ac4C achieved the highest accuracy of 80.03%, with SPE and AUROC values reaching 83.01% and 87.50%, respectively. XG-ac4C attained the highest SEN value of 93.36%, remaining the highest among all models. Leveraging advancements in deep learning technology, our DPNN-ac4C model outperforms the current models in terms of SPE, ACC, MCC, and AUROC. While our SEN remains lower than XG-ac4C, we surpass the iRNA-ac4C model by 2.6% in SPE, 3.86% in ACC, 6.54% in MCC, and 5.22% in AUROC, strongly demonstrating the superior performance and reliability of DPNN-ac4C in identifying ac4C sites.

**Table 3.** Performance comparison with existing methods based on the 10-fold cross-validation of the training set. (Bold indicates maximum value in the current column)

Model	SEN (%)	SPE (%)	ACC (%)	MCC (%)	AUROC (%)
PACES	78.49	77.02	78.56	54.35	84.38
XG-ac4C	**93.36**	54.62	74.07	52.23	83.31
DeepAc4C	80.62	80.56	80.26	60.32	86.41
iRNA-ac4C	77.02	83.01	80.03	60.1	87.5
DPNN-ac4C (ours)	82.18	**85.61**	**83.89**	**66.55**	**92.72**

**Figure 5.**
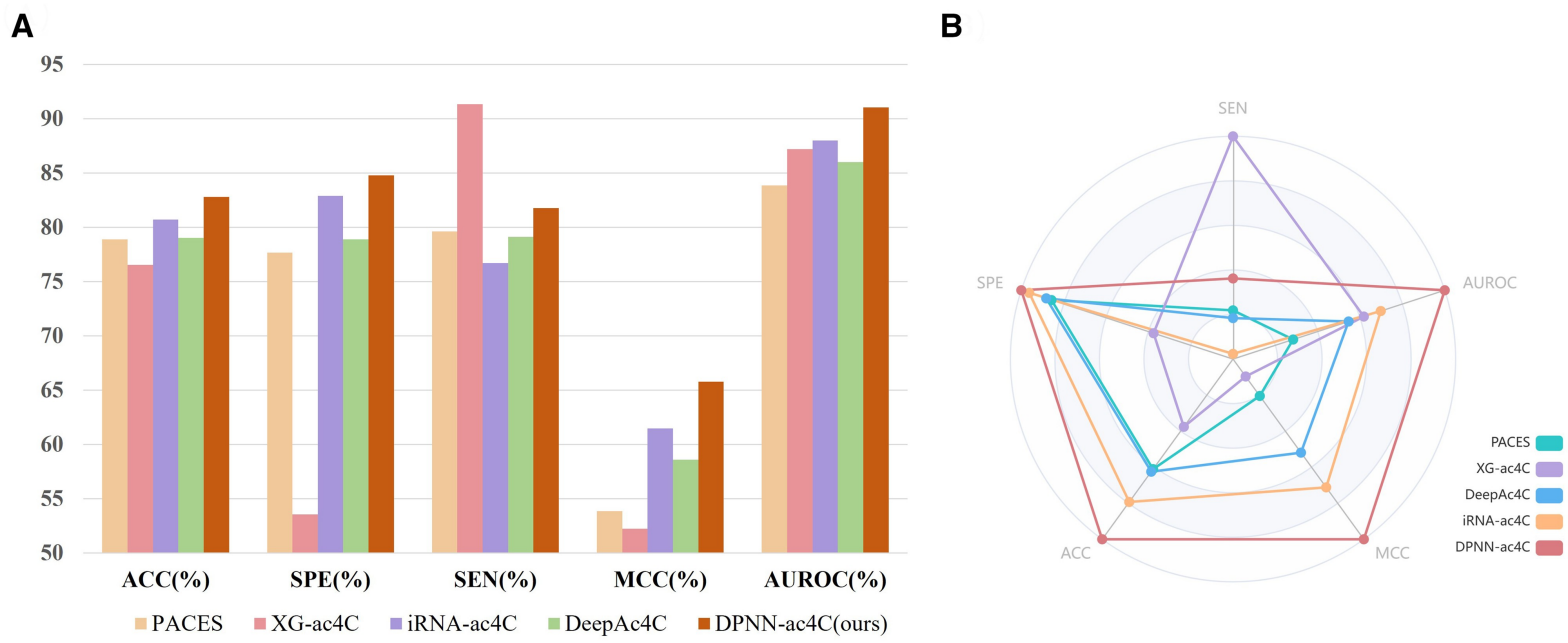
Evaluation metrics on the independent test set for various algorithms. (A) The bar chart comparing the performance of different metrics on the test set for various algorithms. (B) The corresponding radar chart.

**Figure 6.**
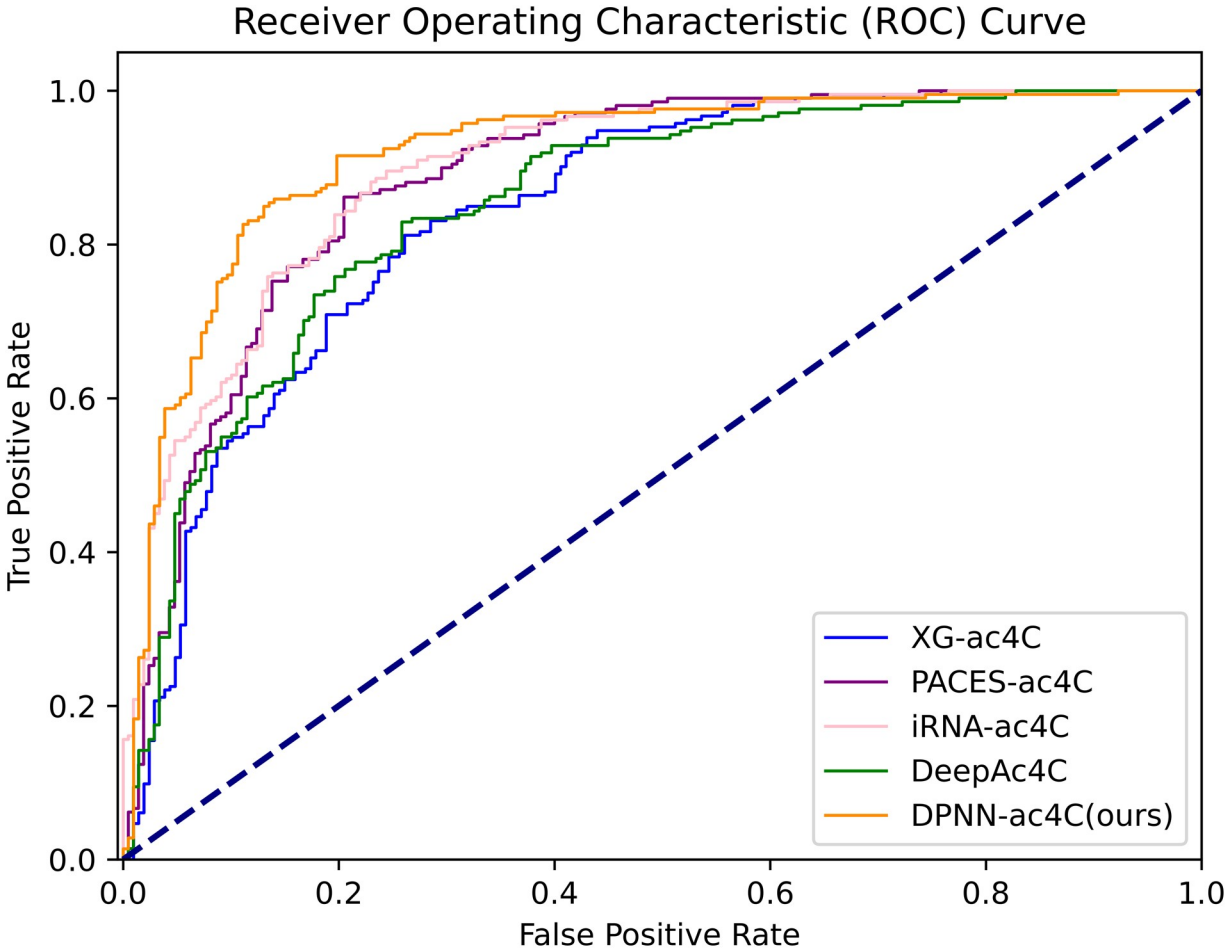
Performance comparison of ROC curves on the independent test set.

To ensure the rigor of the experiment, we also conducted data comparisons on an independent test set. In Table 4 and Fig. 5, it can be observed that all five metrics of our model on the independent test set experienced varying degrees of decline. While our SEN remains lower than XG-ac4C, we surpass the iRNA-ac4C model by 1.87% in SPE, 2.07% in ACC, 4.32% in MCC, and 3.02% in AUROC, strongly demonstrating the superior performance and reliability of DPNN-ac4C in identifying ac4C sites. According to Fig. 6, the ROC curve of DPNN-ac4C is higher than that of existing models, indicating that the proposed model can achieve a higher true positive rate (TPR) at the same false positive rate (FPR). This further validates the superior performance of our model.

**Table 4.** Performance comparison with existing methods based on the independent test set. (Bold indicates maximum value in the current column)

Model	SEN (%)	SPE (%)	ACC (%)	MCC (%)	AUROC (%)
PACES	79.63	77.65	78.89	53.85	83.87
XG-ac4C	**91.35**	53.54	76.54	52.23	87.21
DeepAc4C	79.11	78.89	79.03	58.57	86.49
iRNA-ac4C	76.7	82.91	80.71	61.46	88.01
DPNN-ac4C (ours)	81.78	**84.78**	**82.78**	**65.78**	**91.03**

### 3.4 UMAP visualization

Uniform Manifold Approximation and Projection (UMAP) is a nonlinear dimensionality reduction algorithm utilized for visualizing high-dimensional data by preserving both local and global structures post-reduction. Acting as a manifold learning technique, UMAP transforms high-dimensional data into a lower-dimensional space, facilitating the comprehension and visualization of intricate data patterns. Its significance extends to various data analysis and machine learning tasks (McInnes *et al.* 2018).

**Figure 7.**
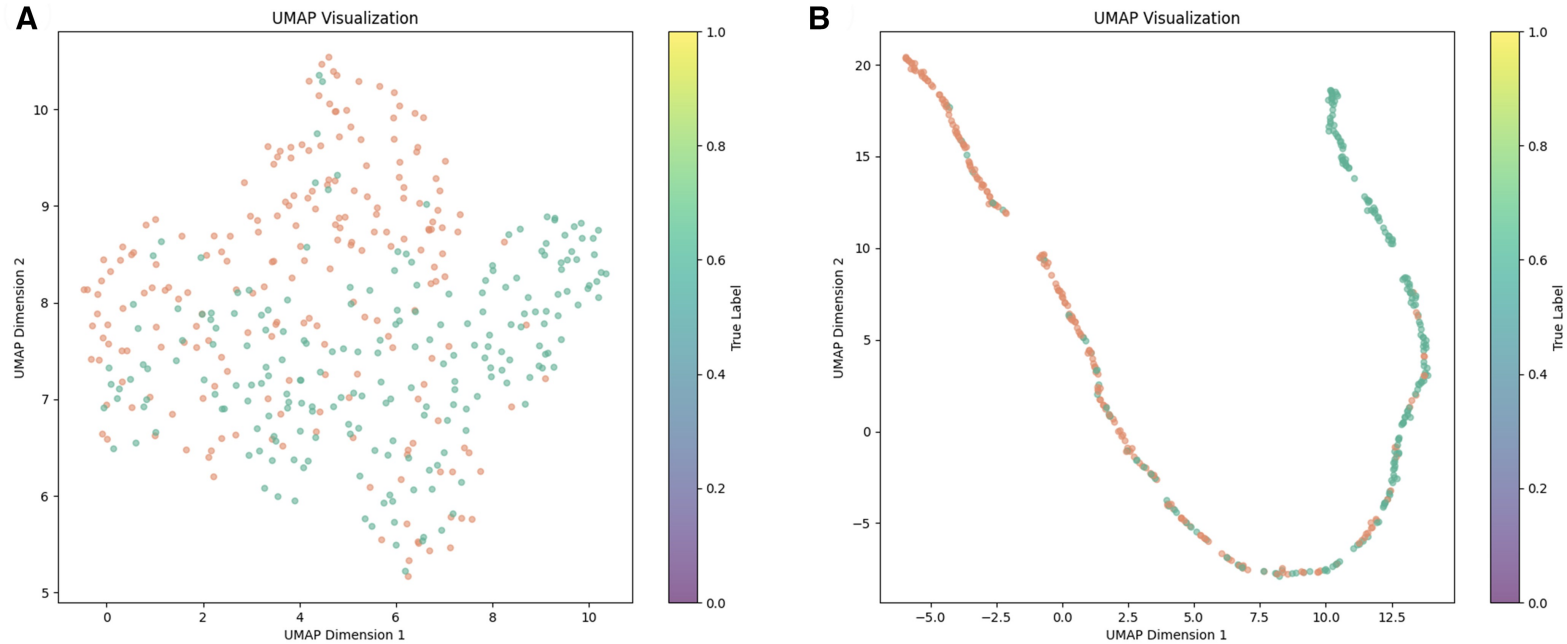
UMAP visualization of sample features before and after DPNN-ac4C training. (A) Depicts the distribution of sample features without DPNN-ac4C training. (B) Illustrates the distribution of sample features after DPNN-ac4C training.

As depicted in Fig. 7, negative sample features are denoted in green, while positive ones are shown in orange. Figure 7A illustrates the distribution of sample features absent DPNN-ac4C training, appearing scattered. Despite the predominance of positive samples in the upper region and negative ones in the lower region, discerning between the two remains challenging. In contrast, Fig. 7B displays the distribution of sample features post DPNN-ac4C training, revealing a more cohesive distribution with distinct boundaries separating negative and positive samples. Consequently, it can be inferred that the DPNN-ac4C model adeptly discriminates between positive and negative samples.

### 3.5 Model robustness evaluation

The results in Table 5 indicate that after FGM attack, the model’s overall recognition accuracy does not significantly decline. It sustains high-precision predictive capability, demonstrating robustness within an acceptable error margin. Hence, we conclude the model’s robustness. This demonstrates that even if there are sequencing errors present in the original RNA sequence, our model can still maintain a high level of accuracy in identifying the RNA sequence’s category.

**Table 5.** Performance comparison with our methods without FGM attack.

Model	SEN (%)	SPE (%)	ACC (%)	MCC (%)	AUROC (%)
Attacked	84.77	83.21	83.73	65.57	92.35
DPNN-ac4C	82.18	85.61	83.89	66.55	92.72

## 4 Conclusion

N4-acetylcytidine (ac4C) is a naturally occurring RNA modification pivotal in post-transcriptional regulation, mRNA stability, and translation efficiency. Understanding its distribution patterns unveils RNA function mechanisms and its impact on the global regulatory network, linked to diseases like cancer (Luo *et al.* 2023). Accurate ac4C site identification aids in discovering biomarkers and therapeutic targets.

The DPNN-ac4C model proposed enhances site identification accuracy by 2.22%. This improvement stems from integrating advanced multi-head self-attention mechanisms. These heads prioritize parts crucial for ac4C site distinction through distinct linear transformations, while the dual-path neural network design captures temporal RNA sequence information and relevant local data separately. Furthermore, the model’s robustness is validated by applying the FGM method.

Despite advancements, our study has limitations. We have not exhaustively tested all manual RNA feature characterizations. In addition, traditional machine learning methods like IFS and GBDT remain valuable for enhancing model interpretability. Future work will explore more RNA representation methods and integrate additional traditional machine learning methods for model refinement. In summary, DPNN-ac4C is a potent tool for identifying ac4C sites, expected to further elucidate their function and biological mechanisms with improved accuracy.

## Data Availability

The model code and dataset are publicly available on GitHub (https://github.com/shock1ng/DPNN-ac4C).
